# Hysterical Earache

**Published:** 1905-10-07

**Authors:** 


### HYSTERICAL EARACHE.

In a lecture on earache, Mr. Richard Lake[1] recognises cases in which this symptom is a manifestation of hysteria, and divides such cases into two classes, adding, however, the preliminary remark that in every case there is some, though it may be very slight, pathological condition of the auditory apparatus. The first form of hysterical earache is found as a severe pain of sudden onset. Even the most gentle examination of the ear elicits extreme tenderness. The skin of the auricle and of the external meatus is highly hyperæsthetic, and an attempt to introduce a speculum appears to cause as much pain as though a furuncle of the meatus were present. Yet inspection of the parts shows all redness and other signs of inflammation to be absent. Nothing abnormal can be detected, except perhaps some evidences of a slight catarrh. The treatment of this variety of hysterical earache requires no special measures except those which apply to the general condition from which the patient is suffering. In the second variety of the disease, diagnosis and treatment are both difficult. It is seen in cases of chronic non-suppurative middle-ear disease, such as is usually accompanied by only a slight amount of pain. The patient's general health is commonly depressed, and there is a more or less marked degree of the hysterical or neurotic temperament. In these conditions any pain becomes magnified to the mind of the patient, who may thus come to believe that pus is present in the ear or in the mastoid process, and may urge an operation as necessary to secure relief. Needless to say, operation in these cases ought not to be granted, though to satisfy the patient on this point is a tedious and thankless undertaking.

[1] Clinical Journal, September 27, 1905.

